# Cardiac Myosin Inhibitors in the Treatment of Hypertrophic Cardiomyopathy: Clinical Trials and Future Challenges

**DOI:** 10.3390/biom15081098

**Published:** 2025-07-29

**Authors:** Arnold Kukowka, Marek Droździk

**Affiliations:** 1 Department of Pharmacokinetics and Therapeutic Drug Monitoring, Pomeranian Medical University, 72 Powstańców Wielkopolskich Avenue, 70-111 Szczecin, Poland; arnold.kukowka@pum.edu.pl; 2 Department of Pharmacology, Pomeranian Medical University, 72 Powstańców Wielkopolskich Avenue, 70-111 Szczecin, Poland

**Keywords:** cardiac myosin inhibitors, hypertrophic cardiomyopathy, mavacamten, aficamten, LVOT obstruction, heart failure, pharmacotherapy

## Abstract

Hypertrophic cardiomyopathy (HCM) is a prevalent and often underdiagnosed genetic cardiac disorder characterized by left ventricular hypertrophy and, in many cases, dynamic left ventricular outflow tract obstruction (LVOTO). The development of cardiac myosin inhibitors (CMIs) represents an emerging therapeutic approach in the pharmacological management of obstructive HCM (oHCM). This review offers an integrated and up-to-date synthesis of the cardiac myosin inhibitor class, with a focus on mavacamten, aficamten, and the broader landscape of emerging agents. It also highlights recent clinical trial outcomes, pharmacokinetic and pharmacogenetic considerations, and potential future directions in therapy. Furthermore, we incorporate the most recent data up to May 2025, including late-breaking trial results and real-world safety findings, aiming to provide clinicians with a practical and comprehensive perspective on this evolving drug class. A narrative review was conducted by systematically searching PubMed, Scopus, Google Scholar, and ClinicalTrials.gov for English-language articles and trials published between January 2016 and May 2025. Keywords included “cardiac myosin inhibitor”, mavacamten”, “aficamten”, “MYK-224”, and “hypertrophic cardiomyopathy.” Inclusion criteria encompassed clinical trials and comprehensive reviews specifically addressing CMIs in cardiac applications. CMIs such as mavacamten and aficamten have demonstrated significant clinical benefits in reducing LVOT gradients, improving exercise capacity, and alleviating symptoms in patients with oHCM. Mavacamten is currently approved for clinical use, while aficamten is in advanced regulatory review. Comparative data suggest potential advantages of aficamten in the onset of action, pharmacokinetic profile, and tolerability. Emerging evidence supports the exploration of CMIs in pediatric populations, heart failure with preserved ejection fraction (HFpEF), and non-obstructive HCM (nHCM), although results are still preliminary. Cardiac myosin inhibitors offer a novel, pathophysiology-targeted approach to managing oHCM. While mavacamten has established efficacy, next-generation agents like aficamten may offer improved safety and versatility. Further long-term studies are needed to clarify their role across broader patient populations.

## 1. Introduction

According to the guidelines of the European Society of Cardiology (ESC), hypertrophic cardiomyopathy (HCM) is diagnosed when an increased thickness of the left ventricular (LV) wall is present, in the absence of secondary causes (such as hypertension or tachyarrhythmias). In order to diagnose HCM in adult patients, the LV wall thickness should be equal to or higher than 15mm in any myocardial segment. For children, LV wall thickness is required to be greater than two standard deviations (SD) from the predicted mean (z-score more than 2) [1].

From a statistical standpoint, it is estimated that 1 in 500 individuals may be affected by HCM. However, this figure may be underestimated, as only 10–20% of cases are currently identified [2]. Recent data suggest that the prevalence of HCM may be approximately 1 in 200 individuals, highlighting a growing clinical problem. More widespread use of echocardiographic diagnostics in the general population is the primary reason for the increased HCM diagnosis [3,4].

For example, in Poland, it is estimated that approximately 180,000 people are affected by HCM. Data on the prevalence of hypertrophic cardiomyopathy in Poland between 2016 and 2020 revealed a yearly increase in newly diagnosed HCM cases, except in 2020, which is attributed to the COVID-19 pandemic [5].

In addition to secondary causes of hypertrophic cardiomyopathy, two primary etiologies of HCM can be distinguished: genetic mutations and diseases that mimic HCM in imaging studies (e.g., echocardiography and cardiac magnetic resonance), referred to as phenocopies. These phenocopies exhibit imaging features similar to those seen in genetically determined HCM but have different underlying causes. They are most commonly either storage diseases, such as Fabry disease and Danon disease, or infiltrative conditions, such as amyloidosis.

Regarding genetic mutations, HCM is most frequently associated with mutations in genes encoding cardiac sarcomere proteins. The most commonly affected genes are MYH7, MYBPC3 and TNNT2. Mutations in MYH7 and MYBPC3 account for approximately 60–70% of HCM cases. In contrast, mutations in TNNT2 represent around 3–5% [1,6,7]

The treatment of hypertrophic cardiomyopathy is a complex clinical challenge. Current therapeutic algorithms distinguish two main treatment pathways, and the choice between them is closely linked to two key aspects: The severity of heart failure symptoms, classified using the New York Heart Association (NYHA) functional classification [8], and the presence or absence of left ventricular outflow tract obstruction (LVOTO).

The parameter used to assess LVOTO is the pressure gradient across the left ventricular outflow tract (LVOT), as measured by echocardiography. A normal LVOT pressure gradient should not exceed 30 mmHg, whereas a value equal to or greater than 50 mmHg is considered hemodynamically significant, leading to marked impairment of blood flow. This threshold (50 mmHg) serves as a critical decision point in both pharmacological and invasive treatment strategies according to current clinical guidelines. Yet, it is the value of 30 mmHg of LVOT gradient that is the value used to differentiate patients into two clinical subtypes: those with obstructive hypertrophic cardiomyopathy (oHCM), characterized by LVOT obstruction, and those with non-obstructive hypertrophic cardiomyopathy (nHCM), in whom obstruction is absent. European Society of Cardiology guidelines underline that most patients in the range of 30 mmHg to 49 mmHg LVOT gradient can be treated according to the non-obstructive hypertrophic cardiomyopathy pathway. Cardiac myosin inhibitors (CMIs) are currently used exclusively in the treatment of oHCM. The LVOT gradient is highly relevant in the context of heart failure symptoms. Its elevation can lead to worsening of heart failure manifestations, such as fatigue, increased dyspnea, and a significant reduction in exercise capacity, due to impeded delivery of oxygenated blood to peripheral tissues [1,9,10,11]. 

**Figure 1:**
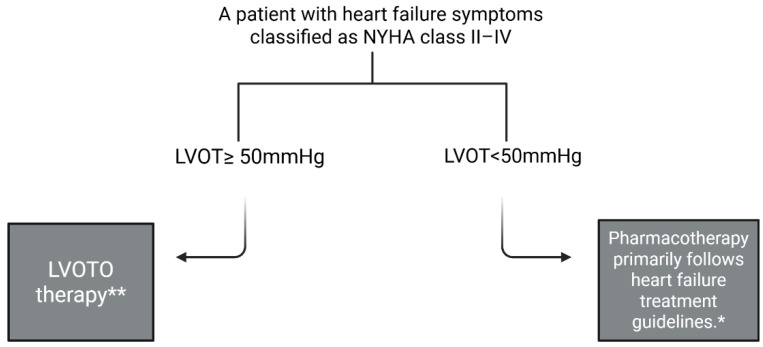
Simplified diagram of the therapeutic pathway for hypertrophic cardiomyopathy based on the guidelines of the European Society of Cardiology [1]. * depending on ejection fraction; ** described in the main text. Created in BioRender: https://BioRender.com/wzb0fkf (accessed on 30 May 2025).

Treatment of LVOTO is guided by the presence of heart failure symptoms. In symptomatic patients with HCM, the first-line therapy is based on beta-blockers. If symptoms persist, non-dihydropyridine calcium channel blockers (such as verapamil or diltiazem) may be added. Subsequently, cardiac myosin inhibitors or disopyramide can be introduced. If symptoms remain refractory to pharmacotherapy, invasive procedures are recommended, either alcohol septal ablation or surgical myectomy (see Figure 1) [1].

The introduction of a new class of medications, i.e., cardiac myosin inhibitors, is a major breakthrough in the treatment of HCM [12]. This article provides a detailed discussion of CMIs. The emergence of this novel pharmacological option not only enables improved symptom control but also contributes to delaying the need for invasive intervention. The development and clinical benefits of CMIs have been acknowledged by the leading cardiology societies, i.e., the European Society of Cardiology (ESC) and the American College of Cardiology (ACC), both of which have issued positive recommendations for their use, in 2023 and 2024, respectively [1,13,14,15].

Currently, the most widely used CMIs is mavacamten, which was approved by the U.S. Food and Drug Administration (FDA) in 2022 [16] and by the European Medicines Agency (EMA) in 2023 [17]. Other agents in this class currently undergoing clinical investigation include the following: aficamten, which has completed Phase III clinical trials [18], and MYK-224, which is currently in Phase II clinical trials [19]. It is noteworthy that, in parallel with the growing population of individuals affected by HCM, there is a steady increase in the number of clinical trials focused on the pharmacological treatment of this condition [20]. Although several recent reviews have focused primarily on mavacamten or aficamten, the present article provides a broader, comparative overview of the entire class of cardiac myosin inhibitors, including mavacamten, aficamten, and investigational agents such as MYK-224 and MYK-581. This review also incorporates the most up-to-date clinical trial data available as of May 2025. In addition, we highlight important aspects such as CYP2C19 pharmacogenetic considerations, regulatory milestones, and the potential future use of CMIs in non-obstructive HCM, heart failure with preserved ejection fraction, and pediatric populations. By addressing these elements, this review aims to deliver a comprehensive and clinically relevant summary of current evidence and future directions in the pharmacological management of HCM with CMIs.

### Physiology of Cardiac Muscle Contraction and Pathophysiology of HCM

Under physiological conditions, cardiac muscle contraction occurs as a result of interactions between myosin filaments (thick filaments) and actin filaments (thin filaments) within a structural unit known as the sarcomere. A key component of myosin is the myosin head, which contains two crucial binding sites: one for actin and one for ATP (adenosine triphosphate). 

**Figure 2:**
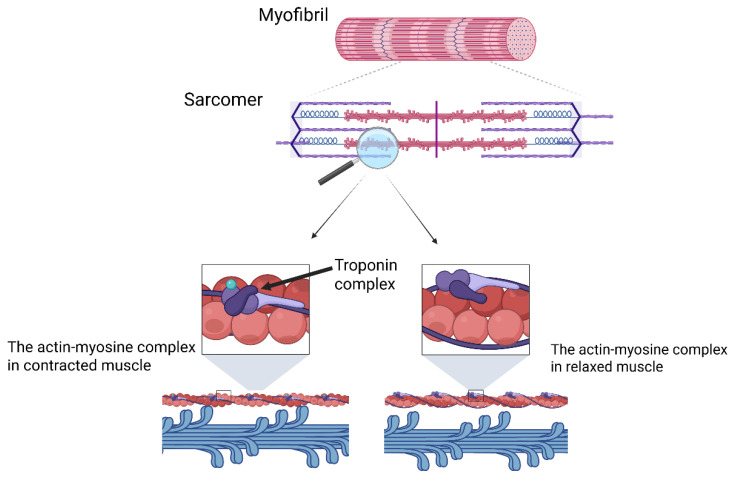
Basic physiology of cardiac muscle contraction: from myofibril structure to sarcomere and actin–myosin interaction. Troponin complex describes the combination of troponin C, I, and T. The colors used in this figure: blue describes myosin (thick filament), red describes actin (thin filament), purple describes tropomyosin, and green describes ion calcium (Ca^2+^). Created in BioRender: https://BioRender.com/y3xg904 (accessed on 30 May 2025).

When ATP binds to the myosin head, it undergoes hydrolysis catalyzed by cardiac myosin ATPase, releasing energy. This energy drives a conformational change in the myosin head, enabling it to bind to actin, forming a cross-bridge. Following the release of inorganic phosphate and then the ADP (adenosine diphosphate), which are products of ATP hydrolysis, the thin filament is pulled toward the center of the sarcomere, resulting in muscle contraction. A new ATP molecule then binds to the myosin head, causing it to detach from actin, allowing the cycle to repeat [21,22]. The basic physiology of cardiac muscle contraction is shown in Figure 2.

**Figure 3:**
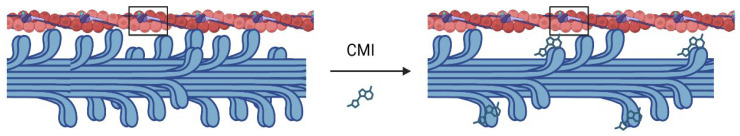
Pathological actin–myosin interaction in hypertrophic cardiomyopathy and its modulation by cardiac myosin inhibitors. The left side of Figure 3 presents the pathological state of cardiac myosin-actin complex in HCM with a larger number of cross-bridges, which results in hypercontraction (as described in the text). The right side shows a state of myosin–actin complex in the presence of CMI: the cardiac myosin inhibitors block excessive myosin heads, and as a result, there is a lower number of cross-bridges. The colors used in this figure: blue describes myosin (thick filament), red describes actin (thin filament), and purple describes tropomyosin. CMI: cardiac myosin inhibitor. The chemical structure shown is symbolic and does not represent any actual compound. Created in BioRender (2025): https://BioRender.com/pie1hc8 (accessed on 30 May 2025).

It is also important to note that, physiologically, two resting states of myosin can be distinguished: the SRX (super-relaxed) state and the DRX (disordered-relaxed) state. These states differ significantly in their ATP consumption rates: SRX consumes far less ATP compared to DRX (0.003 s^−1^ vs. 0.03 s^−1^). Furthermore, the DRX state represents a condition in which the myosin head is primed for activity, i.e., capable of forming cross-bridges and initiating contraction. In contrast, in the SRX state, the myosin head cannot bind to actin, thereby inhibiting contraction [23]. Under pathophysiological conditions, the underlying cause of HCM is often genetic mutations, such as those affecting the β-myosin heavy chain gene (MYH7). This mutation may lead to an increased number of myosin heads in the DRX state, which in turn causes a higher number of cross-bridges between myosin and actin in cardiac sarcomeres [24]. Figure 3 shows the pathophysiological mechanism of HCM. It also includes cardiac myosin inhibitors, which are described more specifically later in this article.

The clinical consequences of the dysfunction include the following: increased myocardial contractility, impaired diastolic relaxation, narrowing of the left ventricular outflow tract (due to increased myocardial fiber mass), and myocardial fibrosis [12,25,26,27].

## 2. Materials and Methods

### 2.1. Data Sources

We searched PubMed, Scopus, and Google Scholar to gather relevant literature. We selected a data frame from January 2016 to May 2025. These platforms were chosen simply because they offer a wide range of peer-reviewed biomedical research and are well-established databases in biomedical sciences. We also took a thorough look at ClinicalTrials.gov to find any relevant clinical trials, mainly 3-phase clinical trials for CMIs.

### 2.2. Search Strategy

The search was performed using a narrative desk review approach. The following keywords and combinations were used: “cardiac myosin inhibitor”, “mavacamten,” “aficamten”, “myosin ATPase”, “hypertrophic cardiomyopathy”, “HCM”, and “hyperertrophic cardiomyopathy treatment”. Boolean operators (AND/OR) were applied to enhance search precision. Only articles published in English were considered. For additional depth, manual screening of reference lists from key articles was performed. The final search was completed on 15 May 2025. 

### 2.3. Inclusion and Exclusion Criteria

Included materials encompassed original research articles, clinical trials, pharmacological studies, and comprehensive review articles specifically addressing cardiac myosin inhibitors. Studies were excluded if they provided only general discussions on sarcomeric proteins without specific reference to pharmacological inhibitors, were editorials, commentaries, or opinion pieces without original data or comprehensive synthesis, or were non-English publications or non-peer-reviewed sources (e.g., blogs or preprints without peer review).

### 2.4. Article Selection and Quality Assessment

Chosen articles were investigated manually for relevance and quality. Emphasis was placed on peer-reviewed studies from reputable journals with transparent methodology and well-described objectives. Clinical trials were evaluated based on the design phase, registration status, and reported outcomes. No formal scoring system was applied, but a qualitative assessment was conducted to prioritize clinically meaningful publications.

### 2.5. Bias Assessment

Bias was minimized through the use of multiple databases and a defined inclusion protocol. Critical source analysis was applied to assess the scientific validity, originality, and contextual relevance of each article. These measures aimed to accurately represent the current state of knowledge on cardiac myosin inhibition.

### 2.6. Transparency and Reproducibility

The methodology employed in this review follows a qualitative narrative approach, ensuring reproducibility through defined search parameters and source documentation. No new experiments, datasets, or protocols were created. All data cited are publicly available or accessible via institutional subscriptions. No ethical approval was required. Generative AI was not used for content generation, data analysis, or synthesis in this manuscript.

## 3. Cardiac Myosin Inhibitors

Cardiac myosin ATPase inhibitors are more commonly referred to in the scientific literature as cardiac myosin inhibitors. They are small-molecule drugs that act by inhibiting a key enzyme responsible for cardiac muscle contraction, namely cardiac myosin ATPase. This inhibition results in a reduced formation of actin–myosin cross-bridges. Additionally, drugs in this class help maintain myosin in its super-relaxed state (SRX). Collectively, these mechanisms counteract the pathophysiological basis of HCM by decreasing myocardial contractility, improving diastolic function, and reducing left ventricular outflow tract obstruction. These effects translate into clinical benefits for patients, including symptom relief, reduction in the LVOT pressure gradient, and improvement in exercise capacity. Importantly, unlike other drug classes used in HCM, CMIs act not only on symptoms but also target the underlying pathophysiology of the disease [28,29,30].

The primary agents in this class include the following: mavacamten (Camzyos ^®^), which currently approved by both the FDA [16] and the European Medicines Agency [17]; aficamten (CK-274), which is currently undergoing clinical investigation [18]; MYK-224, which is in Phase II clinical trials [19]; MYK-581, which is currently in preclinical studies [31]; Blebbastatin, which is used for research purposes [32]. All of these compounds are small-molecule drugs [33]. A summary of the compounds classified as cardiac myosin inhibitors is presented in Table 1.

### 3.1. Mavacamten

**Figure 4:**
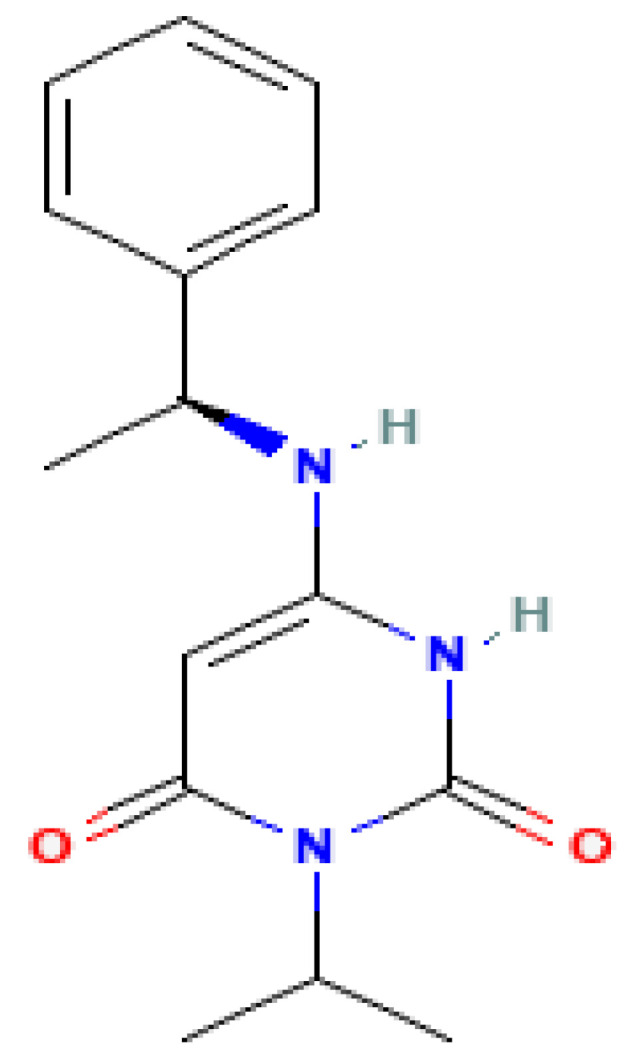
Structure of Mavacamten [34].

Mavacamten, also known as MYK-461, is an allosteric inhibitor of cardiac myosin ATPase and the first approved drug in this class [30], making it also the most extensively studied. The structure of mavacamten is shown in Figure 4, and its molecular weight is 273.33 g/mol [34].

Its mechanism of action, which involves reducing the formation of actin–myosin cross-bridges, has been described previously [30]. From a pharmacokinetic perspective, mavacamten demonstrates high oral bioavailability (>85%), allowing for once-daily oral administration. It has a long half-life, estimated at 6–9 days. Mavacamten is metabolized primarily by two cytochrome P450 isoenzymes: CYP2C19 (≈74%), for which genotyping is recommended by the EMA to identify poor metabolizers [17], and to a lesser extent, CYP3A4 (≈18%) and CYP2C9 (≈8%). Excretion is primarily via the biliary route [35,36]. In preclinical studies on mice, the early administration of mavacamten significantly reduced the progression of hypertrophic cardiomyopathy. Moreover, the same study showed that mavacamten could reverse cardiac hypertrophy in subjects where hypertrophy was already established [37]. In Phase II clinical trials, the PIONEER-HCM study and its long-term extension PIONEER-OLE (*n* = 13) demonstrated that mavacamten significantly reduced LVOT gradients, improved exercise capacity, and enhanced quality of life [38,39]. In Phase III, the EXPLORER-HCM trial (*n* = 251) confirmed its clinical efficacy by showing a mean reduction of 37 mmHg in the post-exercise LVOT gradient (compared to 14 mmHg in the placebo group), improvement in peak oxygen consumption (pVO_2_), an improvement of ≥1 NYHA class in 80% of patients, and a 9.1-point increase in quality-of-life scores versus 5.1 points in the placebo group. Mavacamten demonstrated a safety profile comparable to the placebo [40]. Another clinical trial, The HORIZON-HCM, was conducted on the Japanese population. The study had similar outcomes regarding efficacy and safety of mavacamten when compared to EXPLORER-HCM [41]. 

The MAVA-LTE trial (ongoing through 2029), a long-term extension of EXPLORER-HCM, aims to assess long-term efficacy and safety. Of the 231 patients completing EXPLORER-HCM, 211 were enrolled in MAVA-LTE. The initial dose was 5 mg, with titration based on ejection fraction and LVOT gradient. Preliminary data show sustained LVOT gradient reduction, left atrial volume reduction (~5.5 mL/m^2^), which is a marker of improved diastolic function, at week 180, 66.3% of patients were in NYHA class I, a low discontinuation rate (5.6%), with main adverse effects being atrial fibrillation (14.3%), and worsening heart failure (6.1%) [42]. The VALOR-HCM, an important clinical trial regarding mavacamten and its impact on the treatment of HCM, evaluated its potential to delay or prevent invasive procedures (alcohol septal ablation or surgical myectomy) in patients with NYHA class III/IV of HCM already scheduled for such interventions. At week 16, only 17.9% of patients in the mavacamten group still qualified for invasive treatment, offering a significant clinical benefit [14,43]. However, an important limitation of mavacamten is its potential to reduce left ventricular ejection fraction (LVEF). It is not recommended for patients with LVEF < 55%, and echocardiographic monitoring is essential, especially during the initial treatment phase [17,35]. Following the approval of mavacamten by the FDA and EMA, the number of real-world experience studies evaluating this drug has increased. For example, a study conducted by Ramonfaur et al. [44] in a racially diverse population over an 18-month period demonstrated efficacy and safety outcomes consistent with those observed in clinical trials. The study also found that patients with HCM and obesity were less likely to experience symptom improvement with mavacamten. Another real-world study by Lim et al. [45] demonstrated that mavacamten can be a safe and effective treatment in the Korean population diagnosed with oHCM. Acknowledging mavacamten metabolism, it should be avoided or used with special caution at lower doses in patients co-administered with moderate or strong CYP2C19 or CYP3A4 inhibitors/inducers due to its metabolic profile. Because of the polymorphic nature of the gene coding for CYP2C19, patients should be genotyped for the *CYP2C19* gene to determine the appropriate dose of mavacamten. Patients with a poor metabolizer phenotype may experience up to a threefold increase in mavacamten exposure, which may lead to an increased risk of systolic dysfunction compared to normal metabolizers. If treatment is initiated before the CYP2C19 phenotype is determined, patients should follow the dosing recommendations for poor metabolizers until their CYP2C19 status is known [17,35,46]. This issue has been especially relevant in Asian populations, where the prevalence of poor CYP2C19 metabolism is higher, as observed in the HORIZON-HCM trial (Japanese population) [39], as well as in Chinese [47] and Korean cohorts [45]. In summary, regarding CYP2C19 polymorphism, patients who are poor metabolizers should initiate treatment at a lower dose than normal metabolizers (2.5 mg vs. 5 mg), with subsequent dose adjustment based on echocardiographic imaging [17,46]. The most common adverse events during mavacamten treatment were dizziness, syncope, and atrial fibrillation (AF) [35,36,40]. Due to the occurrence of AF during mavacamten therapy, there is a need for clinical guidelines regarding the safety of anticoagulants and antiarrhythmic (AAD) drugs. A review by Ricci et al. [48] addressed this issue, noting that commonly used anticoagulants, such as vitamin K antagonists and non-vitamin K oral anticoagulants (NOACs), are generally considered safe. However, given the lack of large-scale studies, patients should be closely monitored for potential complications when combining anticoagulation therapy with mavacamten. Regarding AADs, it is important to consider replacing flecainide or propafenone with alternative agents due to their proarrhythmic properties in the HCM population [48,49]. Additionally, mavacamten use has been associated with other arrhythmias, such as premature atrial contractions (PACs), premature ventricular contractions (PVCs), and supraventricular tachycardia (SVT). These arrhythmias were observed primarily at the start of treatment, but the correlation was transient and not present over the long term [50]. To conclude, mavacamten has shown beneficial effects not only on clinical symptoms but also on echocardiographic parameters, with a favorable safety profile. It also offers a non-invasive alternative in treatment pathways.

### 3.2. Aficamten

**Figure 5:**
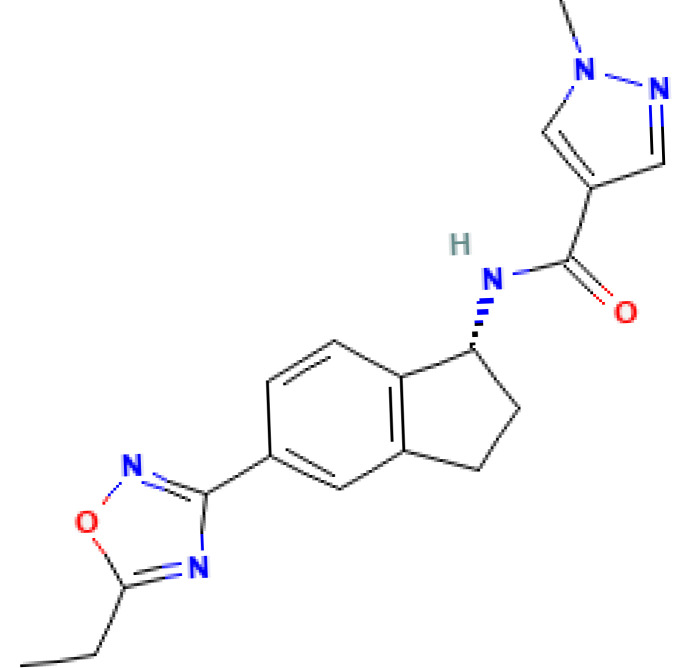
Structure of aficamten [52].

Aficamten, currently under FDA review [51], is a structural analog of mavacamten with a molecular weight equal to 337.4 g/mol (see Figure 5) [52].

Overall, aficamten’s mechanism of action is similar to mavacamten, but it exhibits greater selectivity for the cardiac myosin active site [53]. Thanks to its pharmacokinetic properties, the steady-state drug concentration is set within 2 weeks of treatment, which translates to a faster clinical effect, including NYHA class improvement and LVOT gradient reduction, and is typically achieved in this period of two weeks of treatment [54], compared to 6–9 weeks for mavacamten [40]. Pharmacokinetically, aficamten is metabolized by multiple cytochrome P450 enzymes, reducing the risk of drug–drug interactions and the need for pharmacogenetics testing. It has a half-life of ~4 days, reaches a steady state in ~2 weeks, shows less plasma protein binding (compared to mavacamten), and is renally excreted (~32%), unlike mavacamten, which undergoes minimal renal elimination [55,56,57]. The SEQUOIA-HCM Phase III trial (*n* = 282) demonstrated that aficamten improved pVO_2_ by +1.8 mL/kg/min, reduced resting LVOT gradient by 34.7mmHg, improved NYHA class in 59% of patients, and enhanced overall quality of life [54,58,59]. Earlier trials also showed improvements in LVOT gradient and NYHA class, though some did not reach statistical significance [60,61,62]. In terms of safety, aficamten appears better tolerated than mavacamten. The most common adverse effects of aficamten are dizziness and LVEF reduction below 50%, which occurred in only 3.5% of patients (*n* = 144) after 28 weeks vs. 8.7% in the EXPLORE-HCM study with mavacamten (*n* = 180) [39,52,54,63]. A safety meta-analysis by Davis et al. [64] found that atrial fibrillation occurred in 4.1/100 patients with aficamten vs. 11.5/100 for mavacamten. LVEF-related discontinuation occurred only once for aficamten (*n* = 188), and was transient; in contrast, mavacamten was discontinued completely in eight patients and temporarily in 28 out of 414 [64]. In the context of the cardiac conduction system, aficamten did not prolong the QTc interval and did not influence heart rate [65]. Moreover, regarding aficamten pharmacokinetics and safety, it is worth mentioning that patients with mild to moderate kidney and/or liver impairment may not require dose adjustment, as the pharmacokinetics of aficamten were not significantly different from those observed in individuals with normal liver and kidney function [66]. These findings suggest pharmacokinetic and pharmacodynamic differences between aficamten and mavacamten. Overall, it seems that aficamten acts faster, is cleared more rapidly, and may offer a safer profile, though larger studies are needed. FDA approval is expected by late 2025, and EMA approval by mid-2026 [51,64,67].

### 3.3. Experimental and Emerging CMIs

Blebbistatin is a prototype myosin II inhibitor widely used in experimental research, including studies of muscle physiology and cancer models, due to its fluorescent properties [32,68]. Despite its value as a research tool, its utility in vivo is limited by phototoxicity, poor solubility, and cytotoxicity [69]. To overcome these drawbacks, several derivatives, such as para-nitroblebbistatin and para-aminoblebbistatin, have been developed, offering improved solubility and reduced phototoxicity while retaining inhibitory activity. However, these analogs still exhibit residual cytotoxicity and genotoxicity, limiting their suitability for preclinical development [68,70]. While blebbistatin itself is not clinically viable, its molecular scaffold remains a valuable template for the development of future isoform-selective myosin inhibitors.

Building on these early insights, newer cardiac myosin inhibitors have progressed into clinical development. MYK-224, a structural analog of mavacamten, is currently in Phase II trials. It is designed with a shorter half-life to offer greater dosing flexibility, although detailed preclinical data remain limited [71]. The MERCUTIO trial evaluating MYK-224 in obstructive HCM was terminated for business-related reasons [72]; however, the AURORA-HFpEF trial remains ongoing, assessing its safety and tolerability in patients with heart failure with preserved ejection fraction (HFpEF) [31]. Notably, mavacamten has also shown promise in this population: in the EMBARK-HFpEF trial (*n* = 30), treatment led to a 13% reduction in troponin I and a 26% reduction in NT-proBNP levels, suggesting potential efficacy in HFpEF [73]. In parallel, MYK-581, another CMI analog, is currently in preclinical development and has demonstrated inhibition of HCM progression in animal models [74].

## 4. Discussion

The future of pharmacotherapy for hypertrophic cardiomyopathy using cardiac myosin inhibitors remains filled with open questions. Furthermore, ongoing studies continue to investigate new agents in this class, such as the aforementioned MYK-224 or MYK-581.

To determine the definitive role of CMIs in oHCM, comparative trials with beta-blockers are necessary. The MAPLE-HCM trial, comparing aficamten vs. metoprolol, is completed, but results are pending [75]. However, valuable insights have already emerged from post hoc analyses of existing trial data. In a detailed subgroup analysis of the EXPLORER-HCM and MAVA-LTE trials, Wheeler et al. [76] investigated the impact of background beta-blocker use on the efficacy of mavacamten. Their findings revealed that while the improvement in peak oxygen consumption (VO_2_) was attenuated in patients on beta-blockers due to chronotropic incompetence, the overall clinical benefit of mavacamten was preserved. Key endpoints such as reductions in LVOT gradients, improvements in NYHA functional class, and decreases in NT-proBNP levels were largely unaffected by beta-blocker use. Notably, the data raise the hypothesis that in certain patients, beta-blocker withdrawal or dose reduction could potentially unmask additional benefits of CMI therapy, particularly in terms of exercise capacity. Nevertheless, given the multifactorial indications for beta-blockers in HCM (e.g., arrhythmia management, coronary disease, and comorbid hypertension), this approach must be individualized. The upcoming results from head-to-head trials such as MAPLE-HCM are therefore essential to establish whether CMIs could supplant beta-blockers as first-line pharmacotherapy in symptomatic obstructive HCM.

The role of CMIs in nHCM is under investigation in the ACACIA-HCM trial (aficamten). This is the first aficamten study in the nHCM population [77]. Regarding aficamten, an interesting study was conducted by Masri et al. [78], in which they evaluated the combined efficacy of aficamten administered concurrently with disopyramide, a drug with a well-established role in the treatment of hypertrophic cardiomyopathy. In this study, researchers divided patients from clinical trials of aficamten (REDWOOD-HCM, SEQUOIA-HCM, and FOREST-HCM) into four groups: patients receiving continuous treatment with both aficamten and disopyramide; patients receiving both aficamten and disopyramide, with subsequent discontinuation of disopyramide; patients receiving disopyramide and the placebo; patients receiving disopyramide and aficamten, with subsequent discontinuation of aficamten. The results were very positive for aficamten. The study showed that the combination of disopyramide and aficamten did not demonstrate superiority compared to aficamten alone. Moreover, the discontinuation of aficamten (which left patients treated only with disopyramide) resulted in a higher left ventricular outflow tract gradient, recurrence of symptoms, and NT-proBNP levels returning to baseline levels (before starting aficamten treatment). Thus, the use of aficamten alone may be sufficient to alleviate symptoms of obstructive HCM, potentially allowing for the discontinuation of disopyramide when patients begin treatment with aficamten. In addition, these results may lead to a reduced number of drugs in the patient’s treatment, which offers benefits such as a lower risk of drug interactions and fewer treatment complications. [79,80]. Mavacamten has already been evaluated in nHCM through the MAVERICK-HCM trial [81], which showed improved biomarkers (NT-proBNP and troponin I), but no significant improvements in pVO_2_ or NYHA class, which was limited by the small sample size (*n* = 59), short duration (16 weeks), and reliance on surrogate endpoints. To address these limitations, the ODYSSEY-HCM trial (*n* = 580) was launched in 2022, with extended follow-up through 2029. However, interim results were negative, showing no improvement in pVO_2_ or KCCQ-23 CSS scores, leading to early termination. Investigators suggested that nHCM may represent a distinct disease requiring different pharmacologic strategies [82,83]. A study by Amr et al. [84] also addressed a similar issue, but from a different perspective. Among patients with obstructive HCM, only a portion of this population was included in clinical trials of cardiac myosin inhibitors (CMIs) during phases 2 and 3. According to the authors’ observations, the most common reason for exclusion was an insufficient LVOT gradient. This highlights an important issue, particularly for patients with a resting LVOT gradient in the 30–49 mmHg range [1,84]. For example, in the SEQUOIA-HCM trial (a clinical trial for aficamten), the inclusion criteria were a resting LVOT gradient ≥30 mmHg and <50 mmHg and a post-Valsalva (provoked) LVOT gradient ≥50 mmHg [13]. In contrast, the EXPLORER-HCM trial (a clinical trial for mavacamten) used stricter criteria, requiring a peak LVOT gradient of at least 50 mmHg (either at rest or after Valsalva maneuver/exercise) [40]. These differences raise an important question: from what LVOT gradient should CMIs be considered? Currently, the general consensus is that 50 mmHg serves as the primary threshold. Another key area of CMIs is their pediatric use (<18 years). The CEDAR-HCM trial, currently recruiting, is testing aficamten in this population [85]. Given promising animal model data with mavacamten [35] and aficamten’s potentially superior safety [64], this study may be highly impactful in reshaping HCM therapy. The clinical development of cardiac myosin inhibitors continues to evolve, with next-generation agents such as MYK-224 and MYK-581 under investigation and expanding interest in broader indications (for well-established CMIs such as mavacamten and aficamten), including HFpEF, non-obstructive HCM, and pediatric populations. Despite some setbacks and unanswered questions, ongoing trials reflect a hopeful trajectory for the future of CMI-based pharmacotherapy in hypertrophic cardiomyopathy.

## 5. Conclusions

Cardiac myosin inhibitors (CMIs) reduce myocardial contractility and are currently indicated for a narrow subset of patients with obstructive hypertrophic cardiomyopathy (oHCM). As of now, mavacamten is the only CMI approved for clinical use. Its newer analog, aficamten, may reach the market by late 2025, pending regulatory approval. Both agents have demonstrated favorable clinical and hemodynamic effects, along with acceptable safety profiles in clinical trials. CMIs may expand their therapeutic scope in the future to include pediatric populations and patients with HFpEF or nHCM. However, as a relatively new drug class, CMIs will require further large-scale clinical trials to establish their long-term efficacy, safety, and optimal place in therapy.

## Figures and Tables

**Table 1:** Regulatory status and clinical development stage of selected cardiac myosin inhibitors (references in the text).

Drug Name	FDA Approval	EMA Approval	Clinical Development Stage
Mavacamten	Approved (2022)	Approved (2023)	Available on the market
Aficamten	Pending approval	Pending approval	Phase III completed
MYK-224	Not approved	Not approved	Phase II
MYK-581	Not approved	Not approved	Preclinical phase
Blebbistatin	Not approved	Not approved	Research use only

FDA—Food and Drug Administration; EMA—European Medicines Agency.

## Data Availability

No new data were created or analyzed in this study. Data sharing is not applicable to this article.

## Abbreviations

The following abbreviations are used in this manuscript:

HFpEF	Heart Failure with Preserved Ejection Fraction
HCM	Hypertrophic Cardiomyopathy
oHCM	Obstructive Hypertrophic Cardiomyopathy
nHCM	Non-Obstructive Hypertrophic Cardiomyopathy
CMI	Cardiac Myosin Inhibitor
